# Techno-economic assessment of implementing photovoltaic water villas in Maldives

**DOI:** 10.1016/j.isci.2023.106658

**Published:** 2023-04-11

**Authors:** Lingfei Qi, Yuan Wang, Juhuang Song, Cunhong Yin, Jinyue Yan, Zutao Zhang

**Affiliations:** 1 School of Mechanical Engineering, Guizhou University, Guiyang Guizhou 550025, PR China; 2 Department of Building Environment and Energy Engineering, The Hong Kong Polytechnic University, Hongkong, China; 3 School of Business, Society and Energy, Mälardalen University,SE 72123 Västerås , Sweden; 4 School of Mechanical Engineering, Southwest Jiaotong University, Chengdu 610031, China

**Keywords:** Energy management, Energy Modeling, Energy policy, Energy resources, Energy Systems

## Abstract

Solar energy is considered to be an effective measure to alleviate the shortage of power supply in the Maldives. In this paper, a roof photovoltaic (PV) system integrated into water villas in the Maldives was investigated. Three islands—Ayada Maldives, Angaga Island Resort, and JA Manafaru, located in the southern, central, and northern parts of Maldives—were selected for a case study. The potential of PV installations in Ayada Maldives, Angaga Island Resort, and JA Manafaru reaches 1,410, 445, and 742 kW, with corresponding annual power generation of 2.04, 0.64, and 1.12 GWh, respectively. The profits over the life cycle of 25 years of the above three studied islands are 4.86, 1.52, and 2.90 million USD, respectively, with payback periods in the range of 6–7 years.

## Highlights

• The techno-economics of deploying PV water villas in Maldives are evaluated
• The effects of roof structure on PVs energy production have been studied
• The PV capacity of all water villas in Maldives can reach 86.3 MW
• The total profits over the life cycle are 297.3 million USD

## Graphical abstract

**Figure:**
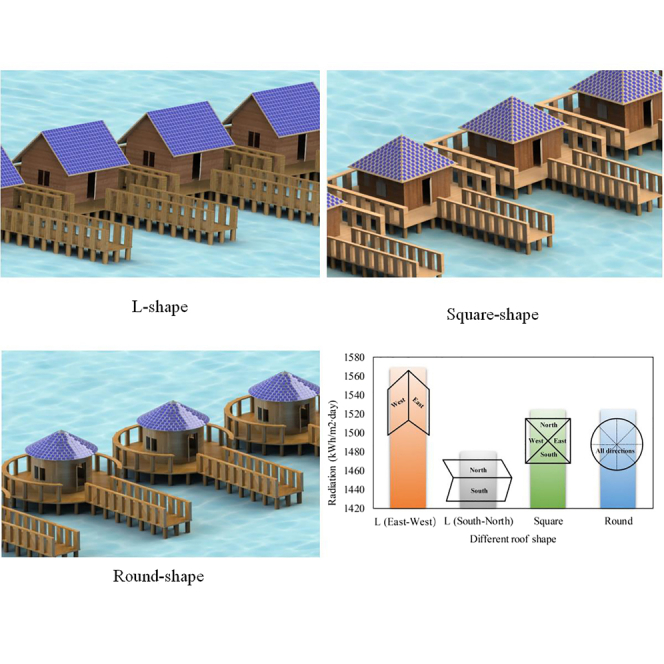

## Teaser

Energy management; Energy Modelling; Energy policy; Energy resources; Energy Systems

## Introduction

In order to promote future sustainability, the integration of renewable energy technologies into buildings is considered an effective solution.^1^^,^^2^ Especially for remote islands, the development of buildings with clean energy can help maintain the local ecological balance.^3^^,^^4^ In recent years, the Maldives archipelago has attracted a large number of tourists due to its beautiful natural scenery and cultural characteristics.^5^ Water villas have served as a unique type of hotel residence, and have been favored by people from all over the world. The vigorous development of tourism has brought huge economic benefits to the Maldives, as well as the costs of increasing energy demand and negative environmental impact. In order to maintain the long-term stability and prosperity of the Maldives, it is necessary to consider the development of environmentally sustainable tourism.^6^ One of the most critical measures is to improve the energy structure and perform energy transition.^7^ Currently, the main source of electricity in the Maldives is still diesel power generation, which will cause direct damage and indirect damage to the environment, including air pollution, greenhouse effect, acid rain, and land erosion.^8^ In addition, diesel needs to be imported, and the transportation cost is extremely high, resulting in the Maldives having the highest electricity price compared to other South Asian regions. Studies have shown that using renewable energy to power the Maldives islands will be an effective measure to protect the environment and reduce energy costs.^9^^,^^10^^,^^11^ It will be significant for the water villas to be integrated with a renewable energy power supply (REPS) system. In this paper, we propose a REPS system for powering the water villas, and investigate its feasibility and technical and economic performances. In order to evaluate the feasibility of REPS for water villas, a crucial factor is the utilization of appropriate renewable energy as the power source. The main renewable resources available in the Maldives include biomass, wave, wind, and solar energy.

Regarding biomass energy, according to the World Bank, bio-waste power generation can be up to 0.33 kg/cap/day. Biomass power generation comes mainly from bananas, but the amount of generation in the Maldives continued to decline from year 2006–2013.^12^ In order to improve the efficiency of standalone biomass power generation, combining some other resources with biomass can be considered for power generation. Ghenai and Janajreh designed a solar-biomass hybrid power generation system which can increase power generation, but the proposed system is complicated and costly.^13^

The second type of renewable energy considered for power supply is wave energy. Beetham and Kench carried out experiments and demonstrated that the wave height on the Maldivian shore can reach 0.99 m, and the period is between 6 and 7 s, indicating that the Maldives has potential for wave energy utilization.^14^ Contestabile et al. pointed out that annual offshore wave power range in the Maldives is between 8.46 and 12.75 kW/m, which lays the foundation for the development of wave energy power generation technology.^15^ Hideki and his team proposed a wave energy converter (WEC) which can generate electricity using breaking waves.^16^ Since the wave energy converter needs to be installed in seawater, its metal components face the possibility of corrosion. In addition, the installation and maintenance of WEC is difficult, causing additionally huge costs.

In addition to biomass and wave energy, the Maldives also has considerable wind energy resources. Liu et al. estimated that Maldives wind speed is about 6.4–6.7 m/s, and wind reserves can be up to 3.8×10^11^ kWh/year at 50 m height in N4.7° regions.^17^ In the project proposal of Wind Energy Project in Himmafushi,^18^ Kaafu pointed out that the average annual wind speed in the Maldives can reach 6 m/s, and the wind energy power density can be up to 350 W/m^2^. Although wind resources are rich in the Maldives, it needs to be harvested effectively in high and remote places, resulting in difficult installation, long-distance power transmission, and costly maintenance.

Besides, since the Maldives is located near the equator, it is a natural solar resource tank. Hassan indicated that the average annual solar irradiation intensity is approximately 5.2 kWh/m^2^/day, and average sunshine hours are about 2784.5 per year in the Maldives.^19^ Therefore, solar power technologies are widely developed in the Maldives, such as hybrid PV systems and standalone PV systems. Jung and his team proposed a hybrid solar PV-diesel energy storage system, and the results showed that this hybrid system is more economical than other power generation systems.^20^^,^^21^^,^^22^ However, this hybrid system will produce polluting emissions like a pure diesel system, and has high operation requirements. For standalone PV systems, it is difficult to build cost-effective centralized photovoltaic power plants in the Maldives due to the decentralization of islands. Therefore, a distributed photovoltaic power generation system based on rooftops is a more suitable option compared to centralized systems. Ihsan evaluated the feasibility of deploying photovoltaic systems on roofs in the Maldives, and the results showed that the annual power generation of rooftop PV systems is between 4.8 and 8.0 GWh on Hulhumalé Island.^23^ World Bank Group has also declared that rooftop solar is a promising solution for improving the environment and economy of the Maldives.^24^ Previous research on solar rooftops in the Maldives has been based on overall potential assessment from a macro perspective, and lacks analysis from the power supply and demand side.

In addition, the floating photovoltaic (FPV) market has expanded at an alarming rate in the past decade, and its global installed capacity has doubled year by year. This growth is possible because FPV plants have many advantages over ground installation plants, which are mainly related to land occupation and energy efficiency.^25^ However, this expansion is limited to freshwater applications, although the offshore environment has great potential. The lack of maturity and harsh environmental conditions have hindered the transition of the FPV technology to the marine environment. Besides, there were no publications on the technical structure analysis and no specific design standards for marine FPVs. For example, researchers found that the warpage of floating photovoltaic cells is one of the most serious problems that may be encountered due to the adverse wave effects.^26^ Moreover, from the perspective of technology and economy, floating photovoltaic systems are suitable for centralized deployment and not efficiency for the Maldives environment where the power demand is very scattered. Based on the above reasons, this paper does not consider adopting floating photovoltaic technology in Maldives.

In particular, the water villas are the largest number of buildings in Maldives and have the largest demand for electricity. It is very valuable to study and analyze the feasibility of the application of photovoltaic roof on the Maldives water villas. In addition, in order to obtain higher solar energy collection efficiency, some researchers have proposed the tracking photovoltaic roof technology based on maximum power.^27^^,^^28^ However, the cost of photovoltaic system based on maximum power point tracking is relatively high, and it is suitable for high-latitude areas. For low-latitude areas such as Maldives, the solar incidence angle changes little within one day, and it is not necessary to adopt solar tracking PV system. Anang et al. proposed a rooftop photovoltaic system based on fixed optimal tilt angle, which can also effectively improve the power generation efficiency of PV system.^29^ Based on the above principle, the PV rooftop of water villas in Maldives can also adopt the fixed installation method with optimal inclined angle. However, the rooftop structures of water villas are diverse. It is necessary to analyze and compare the performance of PV systems deployed on water villas with different roof structures, so as to obtain the best rooftop structure suitable for deployment of fixed PV system.

Through a comparison of the potential installation and maintenance of various renewable energy technologies, we propose a PV rooftop system for powering water villas in Maldives. The feasibility of the studied system will be analyzed from the power supply and demand side of the water villas. It is well known that economic benefit and grid parity are the driving forces behind the promotion of solar PV systems.^30^^,^^31^ Therefore, the technical and economical performances of the proposed system will be discussed under different solar panel configurations. Three typical islands covered by water villas—namely, Ayada Maldives, Angaga Island Resort, and JA Manafaru—are selected for case study.

The rest of this paper is organized as follows. In results section, we present the methodology of the proposed system, including the overview of photovoltaic water villas, case study, self-consumption estimation, and technical and economic analysis. The results and discussion of self-consumption estimation, and technical and economic estimation of the proposed photovoltaic water villa rooftop are provided in discussion section. Finally, some conclusions are given in the last Section.

## Results

### Overview of the photovoltaic water villa

**Figure 1:**
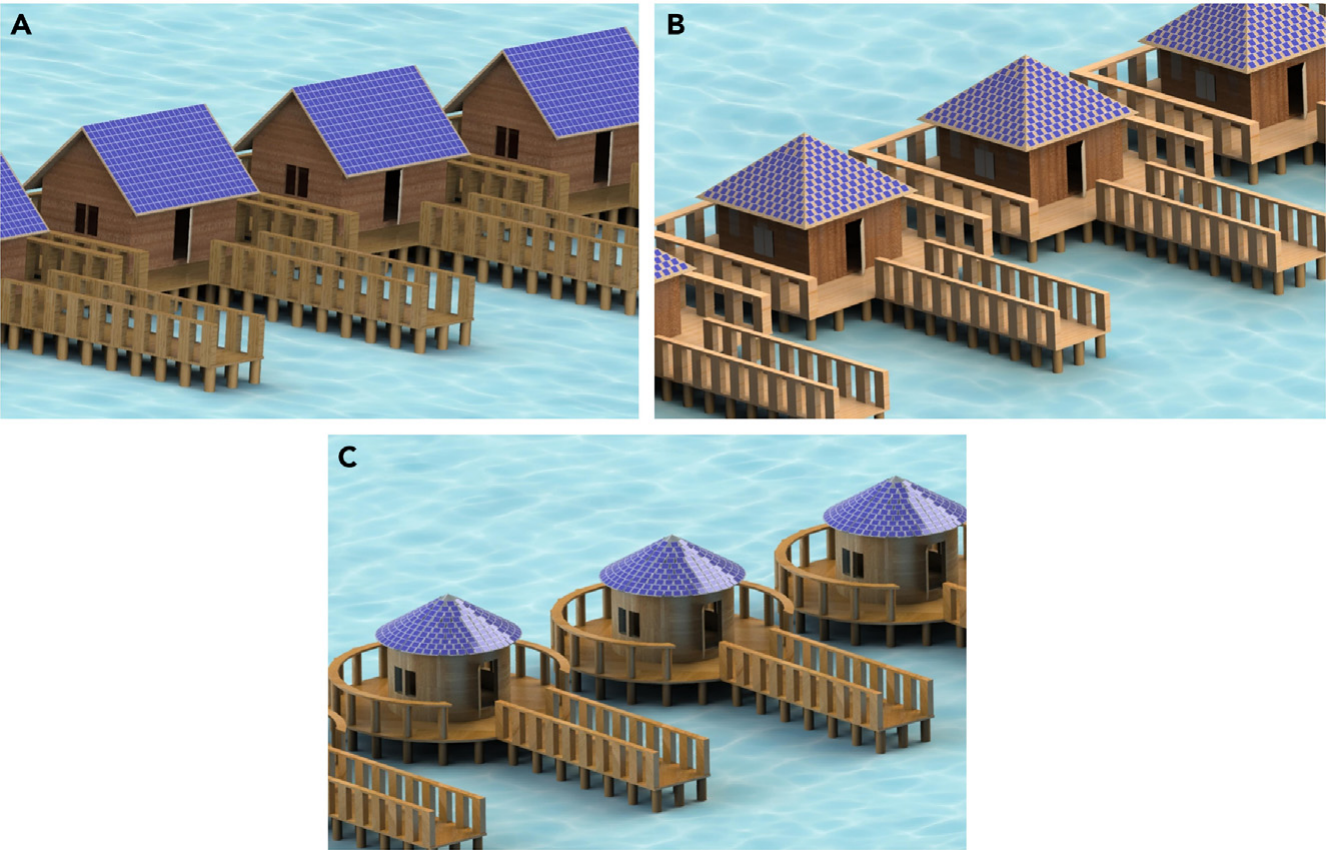
The architecture of the water villa with PV rooftop (A) L-shape. (B) Square-shape. (C) Round-shape.

Developing the solar power technology for water villas not only has the potential to promote sustainable tourism for islands but also has the possibility to be more attractive to tourists. In this paper, we propose a self-powered water villa with PV rooftop. Water villas with three typical roofs—L-shaped roof, Square-shaped roof, and Round-shaped roof—are selected to be integrated with photovoltaic systems, as shown in Figure 1. From the appearance of the 3D model, it is clear that regardless of the roof type deployed with the PV system, the new water villa can offer excellent aesthetics.

### Case study

**Figure 2:**
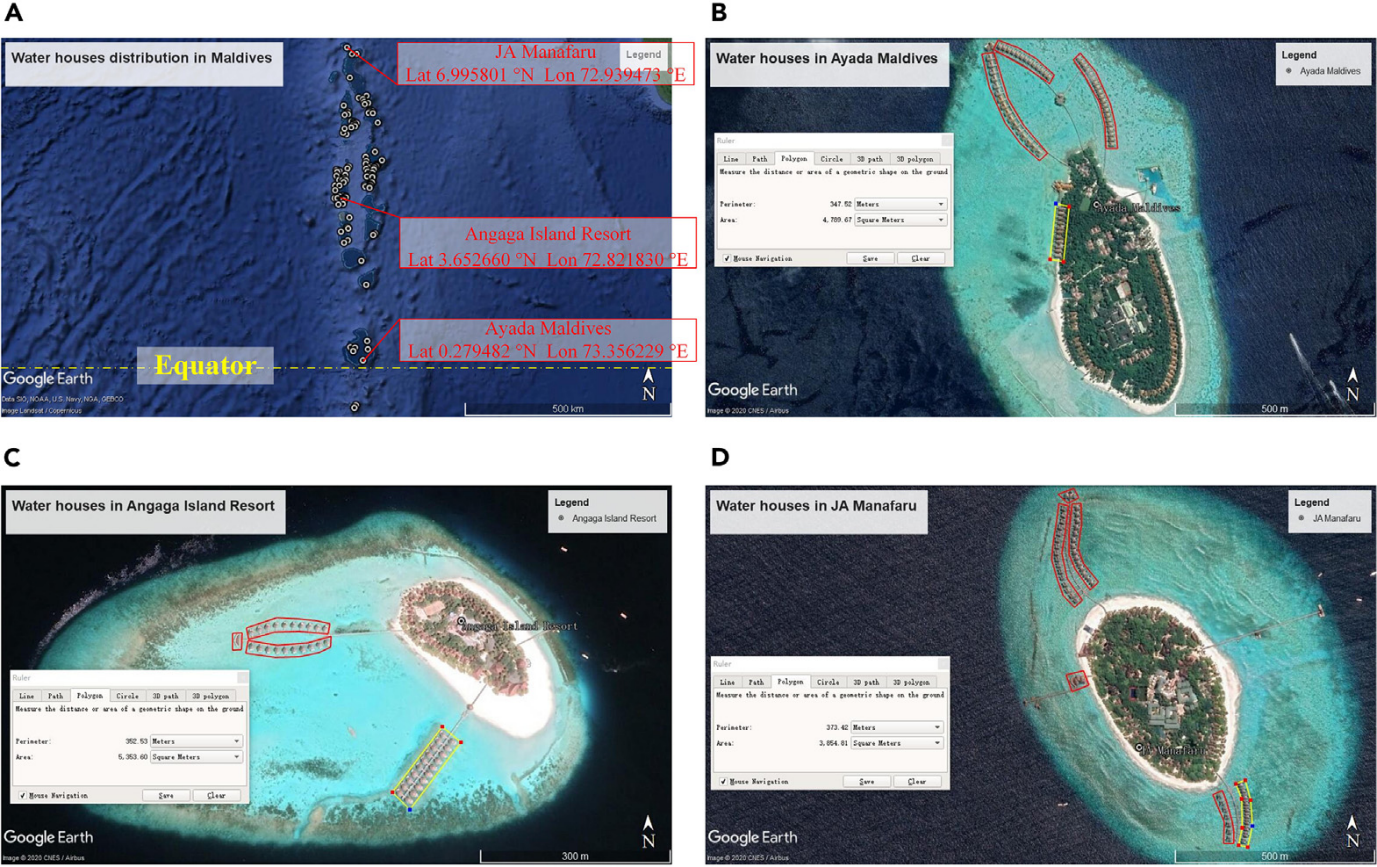
Water villa area measurement using Google Earth’s ranging tool (A) Water villas distribution in Maldives. (B) Ayada Maldives. (C) Angaga Island Resort. (D) JA Manafaru.

As shown in Figure 2A, water villas are spread throughout the Maldives from south to north. In this paper, three typical islands covered by water villas—namely, Ayada Maldives, Angaga Island Resort, and JA Manafaru—are selected for a case study. These studied islands are located in the south, central, and north parts of the Maldives, representing distinct solar power generation potential at different latitudes. It can be seen from Google Earth satellite images that the water villas on these three islands account for a large proportion of the total buildings, as shown in Figures 2B–2D. In order to evaluate the photovoltaic potential of the water villas, the area of the water villas was measured using Google Earth’s ranging tool.^32^ The area of a single water villa, number of water villas, and total area of water villas are listed in Table 1. In this study, flexible solar panels are selected as solar cells, and their parameters are shown in Table 2. The number of solar panels deployed on the all water villa roofs on the three islands is 14097, 4446, and 7423, respectively.

**Table 1:** The area and number of water villas on the three selected islands

	Ayada Maldives	Angaga Island Resort	JA Manafaru
Average area of single water villa (m^2^)	145	67	130
Number of water villas	63	43	37
Total area of water villas (m^2^)	9135	2881	4810

**Table 2:** The parameters of the selected solar panels

Items	Parameters
Solar Cell	Monocrystalline silicon
Dimensions	1200 mm × 540 mm × 2 mm
Area	0.648 m^2^
Maximum power at STC (P_max_)	0.1 kW

STC, Standard Test Condition.

**Figure 3:**
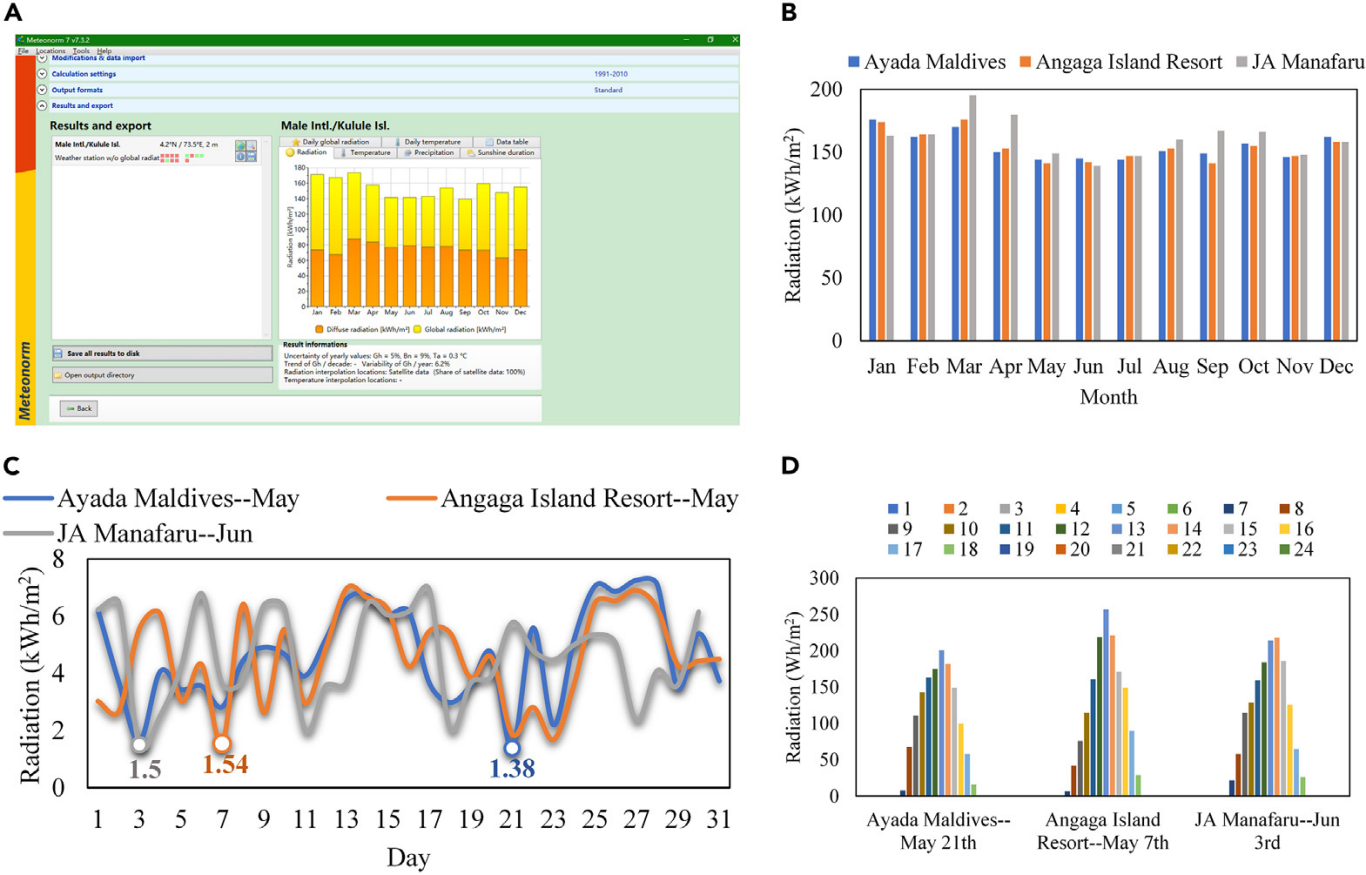
Sunshine conditions in three islands of Maldives (A) Operation interface of Meteonorm. (B) Monthly radiation. (C) Daily radiation. (D) Hourly Radiation.

In order to obtain the solar radiation in the Maldives, we use Meteonorm, which is a unique combination of reliable data sources from Global Energy Balance Archive Data (GEBA) and sophisticated calculation tools.^33^ Figure 3A shows the operation interface of Meteonorm. Sunshine in the Maldives is depicted in Figures 3B–3D. From Figure 3B, we can see that the radiation in different months differs slightly in Ayada Maldives and Angaga Island Resort. In JA Manafaru, the variation in monthly radiation is more obvious. The annual radiation on the three selected islands is 1859, 1853, and 1939 kWh/m^2^. The minimum monthly radiation on the three islands occurs in May and June, 144, 141, and 139 kWh/m^2^, respectively. As shown in Figure 3C, the lowest daily radiation in the months with the weakest radiation is 1.38, 1.54, and 1.50 kWh/m^2^, respectively. The hourly radiation changes in the three days with lowest radiation are depicted in Figure 3D.

### Self-consumption analysis

Recent research indicates that self-consumption of photovoltaic-generated electricity is more economical than transforming it to the grid.^34^ In this paper, we compare the power generation of the PV roof and the power consumption of the water villa to analyze the self-consumption of PV-generated electricity. The parameters of the electric facilities in a water villa in Ayada Maldives are listed in Table 3. The energy consumption of a refrigerator is related to the frequency of opening and closing the refrigerator door. Currently, the daily power consumption of an ordinary refrigerator is about 1 kWh. The total power consumption is 11.64 kWh/day in a water villa located in Ayada Maldives. Assuming that power consumption is proportional to roof area, the load power consumption of a water villa on the other two islands can be calculated, with the results of 5.38 and 10.44 kWh/day, respectively. Assuming that the PV water villa roof is an off-grid system, the system must meet users’ basic electricity demands, even during the weakest solar radiation. Daily power generation at the lowest radiation is calculated to evaluate whether the power generated by a PV roof can meet the self-consumption of a water villa.

**Table 3:** The parameters of the electricity load in a water villa

	Rated power (W)	Number	Working hours (h)	Power consumption (kWh)
Lamp A	65	2	4	0.52
Lamp B	60	4	4	0.96
Lamp C	55	1	4	0.22
TV	120	1	4	0.48
TV signal receiver	35	1	4	0.14
Refrigerator∗	120	1	24	1.00
Computer	80	1	4	0.32
Air conditioner	1000	1	8	8.00
**Total power consumption of one day**				11.64

Refrigerator∗: The power consumption of the refrigerator is related to the frequency of opening and closing the door.

### Energy storage system

Maldives is a group of islands, and the most sub-islands are very scattered, resulting in non-concentrated power demand, so it is not suitable for centralized power supply with photovoltaic grid connection strategy. In addition, the intensity of solar radiation fluctuates greatly over time. In order to improve the power supply stability of PV systems, this paper considers involving the energy storage system to store the electrical energy generated by the water villa PV system. Some related research has shown that lithium-ion batteries, super-capacitors, and flywheel energy storage technologies show good prospects in storing solar energy for building power supply.^35^ However, the leakage current of super-capacitors is large, which is at the mA level; the capacity loss in a single day may exceed 3%. Secondly, if the installation position of the super-capacitor is not reasonable, it is easy to cause electrolyte leakage, which will damage the structural performance of the capacitor. For flywheel energy storage systems, the energy density is not high enough and their self-discharge rate is high. If charging is stopped, the energy will be depleted within a few to dozens of hours. Besides, high-speed running flywheel has significant safety hazards. Lithium-ion battery has strong chemical energy storage stability, so it has excellent capacity retention capabilities. Generally, monthly capacity loss rate can be controlled within 3% for lithium-ion battery. Therefore, this paper uses lithium-ion batteries as the energy storage system for photovoltaic water villas.

## Discussion

### Self-consumption estimation

**Figure 4:**
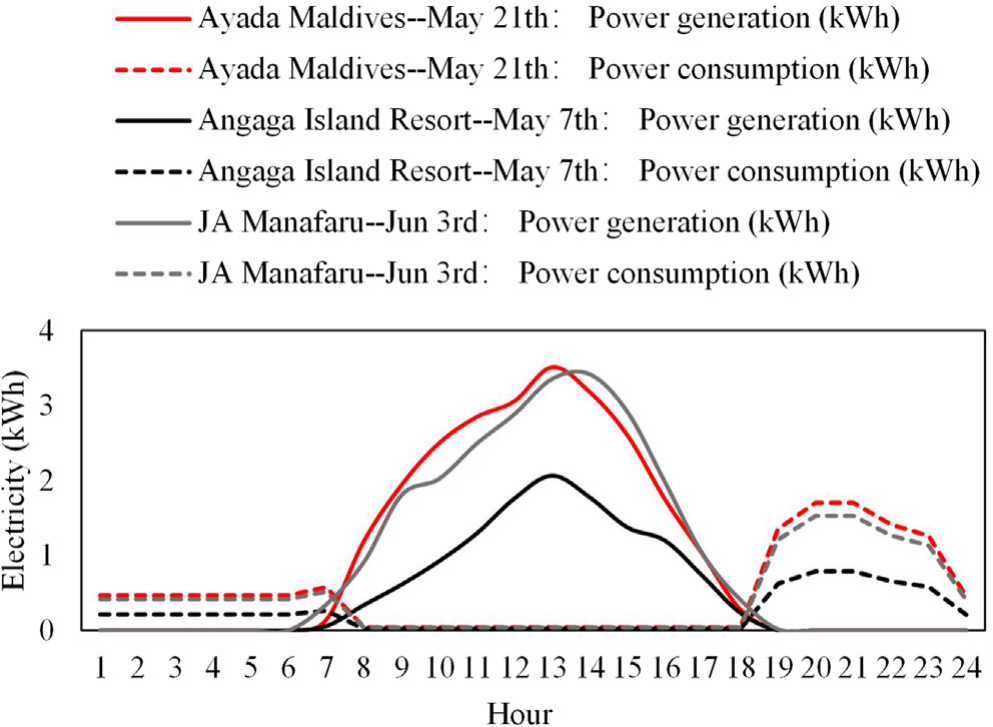
Photovoltaic power generation vs. load consumption under the worst radiation state

Assume that the roof of the water villa is completely covered with photovoltaic panels. The following can be obtained from Figure 3D: the lowest daily radiation on the studied islands is 1.374, 1.537, and 1.503 kWh/m^2^, respectively. According to Equation 2, the daily power generation of a water villa is 24.00, 12.35, and 23.56 kWh, respectively, which is higher than the power consumption of a water villa. The comparison of the hourly photovoltaic power generation to the load power consumption of a single water villa is shown in Figure 4. It can be seen from the figure that the PV roof generates electricity when the load is not working. Therefore, it is recommended to equip a storage system for the PV roof. The storage system not only can supply power to the loads when the PV roof is not in generating state but also can store surplus electricity.

### Technical and economic estimation

**Figure 5:**
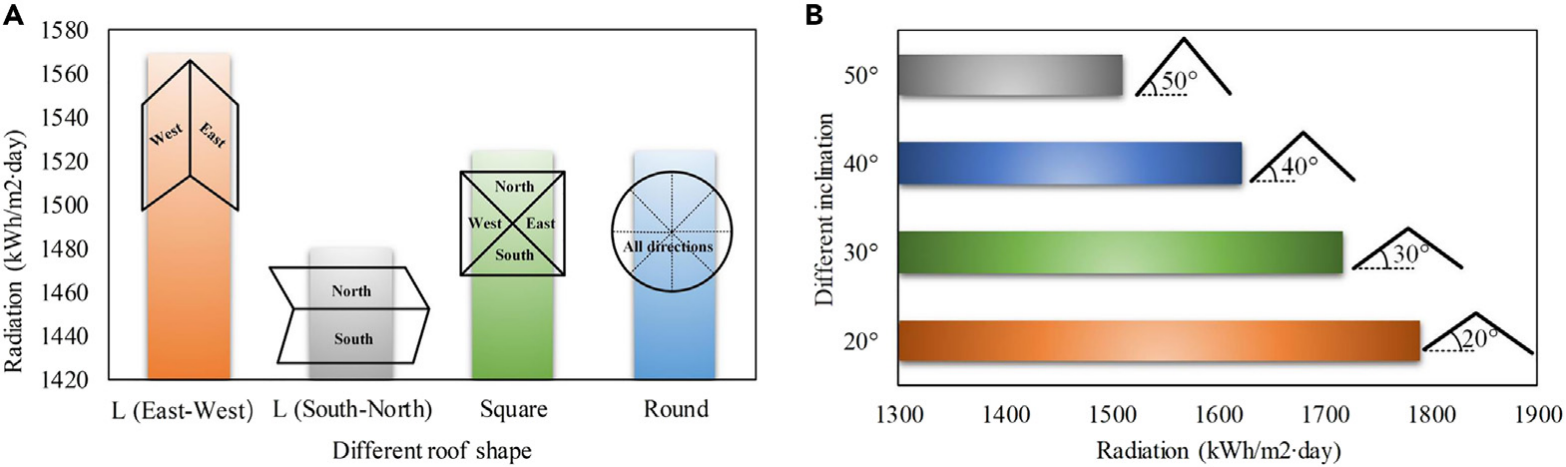
Annual solar radiation received on the PV roofs with different shapes and inclinations (A) Comparison among different shapes. (B) Comparison among different inclinations.

When the PV panel is deployed on an L-shaped roof, it has only two orientations. In this paper, it is assumed that the orientation of the L-shaped roof has two cases: one oriented in the north-south direction and the other oriented in the east-west direction. PV panels installed on Square-shaped roofs have four orientations: east, south, north, and west. PV panels installed on Round-shaped roofs are evenly distributed toward all directions. Figure 5A shows the annual solar radiation received on the PV roofs with different shapes, given the same roof area and geographic location. From this figure, it can be concluded that the L-shaped roof oriented toward east and west has the best technical performance. Besides, the roof has different inclinations varying between 20° and 50°. We compare the technical performance of the east-west L-shaped roof under different inclinations, with the results shown in Figure 5B. It can be seen from this figure that smaller tilt angle corresponds to better technical performance. Therefore, it is recommended to deploy more east-west L-shape water villas with small-inclination roofs for the islands in the future. For the shape selection of photovoltaic systems, this paper believes that the shape of the photovoltaic system needs to match the roof structure. The rectangular photovoltaic system is suitable for the L-shaped roof described in the paper, the square photovoltaic system is suitable for the S-shaped roof, and the circular photovoltaic system is suitable for the R-shaped roof. After theoretical analysis, deploying the photovoltaic system on the L-shaped roof in the east-west direction has the greatest energy benefit, so adopting the rectangular photovoltaic system is the most appropriate.

**Figure 6:**
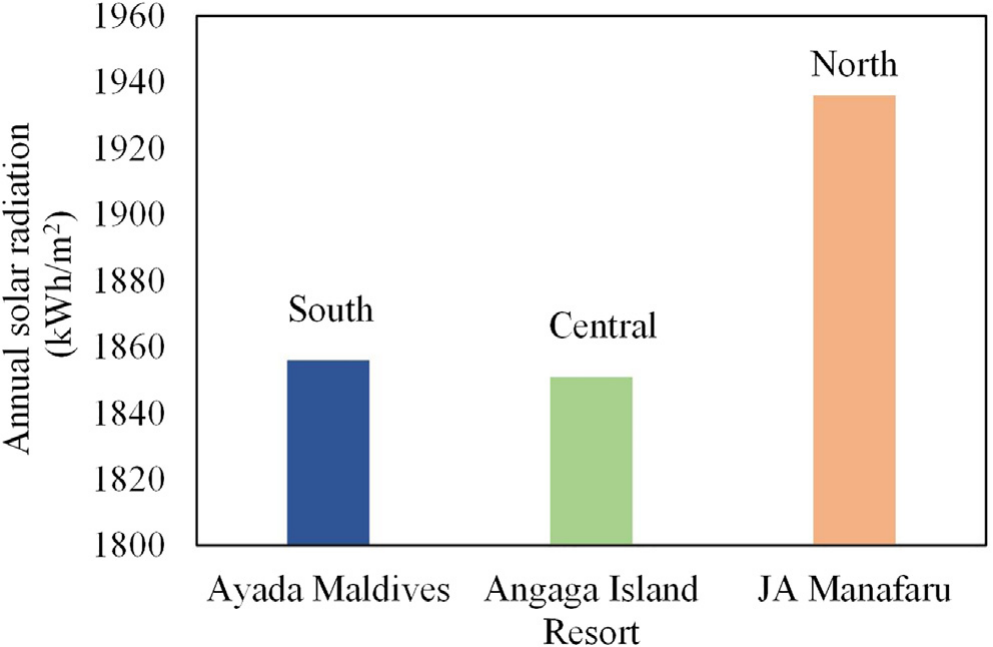
Annual solar radiation on the three islands

In addition to the structure and orientation, the location of the water villas will also affect the technical performance of the PV roof. Since the Maldives islands are generally distributed in the north-south direction, three islands located respectively in the south, central, and north parts of the Maldives are selected for a case study. According to section results, annual solar radiation on these three islands is as shown in Figure 6. From this figure, it can be seen that the northern part of the Maldives has the best solar resources compared to the other two regions. The central and southern parts have similar sunshine conditions. According to Figure 2A, water villas are mainly distributed in the central and northern parts of the Maldives. Therefore, it is recommended to deploy PV water villas in the central and northern Maldives.

**Figure 7:**
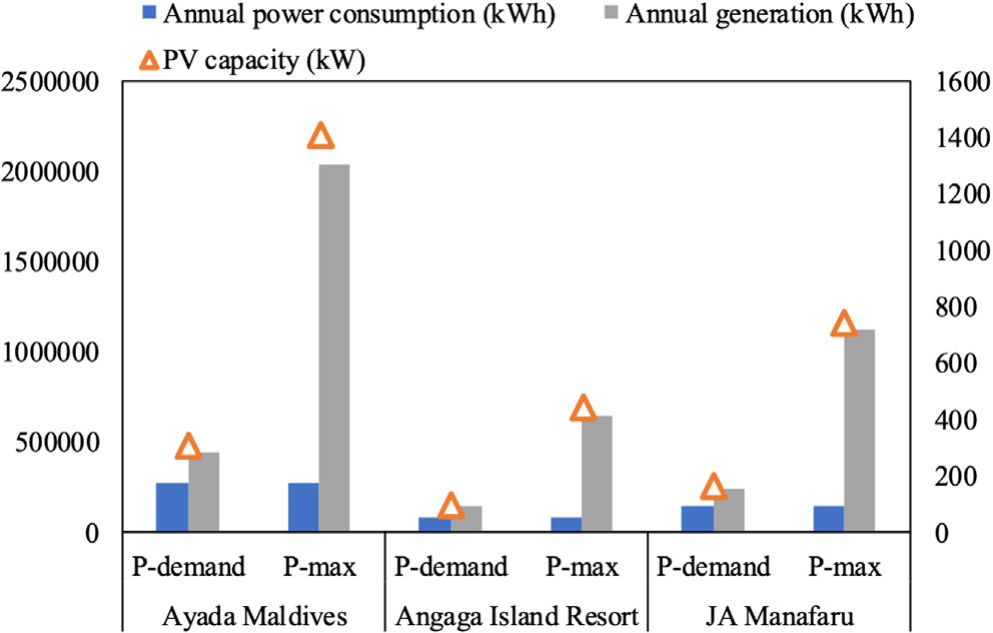
PV capacity, annual power consumption, and annual power generation of the water villas

**Figure 8:**
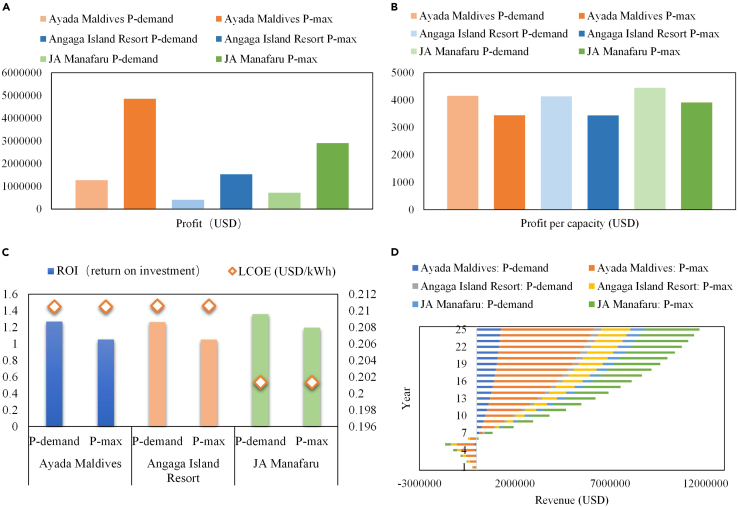
Economic performance of the PV roofs in the life cycle (A) Total profit. (B) Profit per capacity. (C) ROI and LCOE. (D) Revenue and payback period.

According to The World Bank, the cost of photovoltaics and diesel power in the Maldives is 21–25 and 30–40 cents/kWh, respectively. In this case, 25 and 40 cents/kWh are considered as the cost of the PVs installed on water villa roofs and diesel power, respectively. The PV electricity price can be obtained from the actual photovoltaic projects in the Maldives, and comes out to 0.21. The residential electricity price and commercial electricity price in the Maldives can be found in the Maldives Power Industry Investment Prospects and Risk Analysis Report. In order to obtain better energy and economic benefits, two schemes for configuring photovoltaic panels are compared. The first solar panel configuration scheme is to only satisfy the power requirements of the load (P-demand). The second scheme is to reach the maximum power capacity of the water villa roof (P-max). Figure 7 depicts the PV capacity, annual power consumption, and annual power generation of the water villas under the proposed two schemes. From this figure, it can be seen that no matter which configuration scheme is adopted, the annual solar power generation is higher than the annual power consumption of the load on the three studied islands. Therefore, it is recommended that the electricity generated by the PV roofs is first used for powering the water villa, and then the surplus electricity is fed into the grid. The economic benefits of the PV roofs under the proposed electricity utilization mode are shown in Figure 8. By comparing Figures 8A and 8B, it can be seen that although the profit of the P-demand scheme is much lower than that of the P-max scheme, its profit per capacity is higher. From Figure 8C, the return on investment (ROI) of the P-demand scheme is higher than that of P-max. As for the levelized cost of energy (LCOE), it is only related to the location. In the northern and central regions with higher solar radiation, the LCOE of solar electricity is lower. As shown in Figure 8D, although the payback period of the P-max scheme is longer than that of P-demand, more revenue can be achieved during the life cycle of PV panels under the P-max scheme. Taking all the energy and economic results into consideration, it is recommended to adopt the P-max scheme and arrange photovoltaic panels accordingly, assuming sufficient investment capital is available. Thus, the capacities of PV installations reach 1410, 445, and 742 kW, with corresponding annual power generation of 2.04, 0.64, and 1.12 GWh in Ayada Maldives, Angaga Island Resort, and JA Manafaru, respectively. The profits over the life cycle are 4.86, 1.52, and 2.90 million USD, respectively. The ROI on the three islands is 1.05, 1.05, and 1.19, with payback periods of 7, 7, and 6 years, respectively.

According to statistics, there are at least 103 islands with water villas in the Maldives. As calculated by Google Earth, the total area of all water villas is about 558,840 m^2^ Figure 2A shows that the water villas are mainly distributed on the islands at latitude 3.65°N. Based on the estimation methods in the three studied cases, the PV capacity of all water villas in the Maldives can reach 86.3 MW, with corresponding annual power generation of 124.6 GWh. The total profits over the life cycle are 297.3 million USD, with a corresponding payback period of 7 years. Besides, the use of photovoltaic power generation instead of diesel power generation will reduce carbon dioxide emissions. According to previous research, saving 1 kWh of coal-fired electricity will reduce 0.997 kg of CO2.^36^ When the water villas are equipped with photovoltaic roofs, the annual carbon dioxide emissions will be reduced by 124,226 tons. This will greatly contribute to the sustainable development of the Maldives.

### Conclusions

In this paper, a PV system integrated with water villas in the Maldives was studied in three different locations: Ayada Maldives, Angaga Island Resort, and JA Manafaru. The PV rooftop system can reach 100% self-supply of electricity for the water villas. Among different layouts of water villas, the water villas with L-shaped roofs have the optimal technical and economic performance. Compared to other locations, the water villas in the northern regions of the Maldives have the best technical and economic performance. Compared to the P-demand photovoltaic configuration, the P-max configuration achieves higher energy and economic benefits. The PV capacity of all water villas in Maldives can reach 86.3 MW, with corresponding annual power generation of 124.6 GWh. The total profits over the life cycle are 297.3 million USD, with a corresponding payback period of 7 years. Based on the above new findings, it can be concluded that deploying photovoltaic systems on water villa roofs in the Maldives can bring huge energy and economic benefits, reduce the pressure on electricity demand, and promote sustainable development of the tourism industry. Considering their superior technical and economic performances, it is recommended to mainly deploy photovoltaic water villas with L-shaped roofs in the north-central part of the Maldives in the future. In addition, as a unique kind of scenery, photovoltaic water villas have the possibility of attracting more tourists.

### Limitations of the study

This paper focuses on the technical and economic potential of the studied PV water villas in Maldives. In order to actually achieve zero energy in the water villas, the following work will be further conducted in the future: (a) develop an appropriate energy storage system for the PV water villa; (b) design an efficient and flexible control system. If the above two challenges are overcome, the PVs has the possibility to be successfully integrated into the water villas.

## STAR★Methods

### Key resources table

**Table:** 

REAGENT or RESOURCE	SOURCE	IDENTIFIER
**Software and algorithms**		

Meteonorm 8	Meteonorm	https://meteonorm.com/en/
Microsoft Visio 2016	Microsoft Visio	https://www.microsoft.com/zh-cn/microsoft-365/visio/flowchart-software
Microsoft Excel 2013	Microsoft Excel	https://www.microsoft.com/zh-cn/microsoft-365/excel

### Resource availability

#### Lead contact

Further information and requests for resources should be directed to and will be fulfilled by the Lead Contact, Jinyue Yan (jinyue.yan@mdh.se).

#### Materials availability

Not applicable.

### Method details

#### Technical and economic analysis

The potential photovoltaic capacity of the water villa rooftop (*C*) can be calculated by:

(Equation 1) C=N×P

where *P* is the maximum power of one solar panel (kW), and *N* is the number of solar panels.

Annual PV output can be expressed as:

(Equation 2) $$E_{p1}=H_{A}\times \frac{C}{E_{s}}\times K$$

where *E*_*p1*_ is the PV output (kWh) in first year, *H*_*A*_ is local horizontal irradiance (kWh/m^2^), *E*_*S*_ is the standard test condition of PVs (1 kW/m^2^), and *K* is the overall performance coefficient, which is 0.78 in this study. In general, the life cycle of solar panels is 25 years. Therefore, the total PV output during the life cycle (25 years) can be obtained by^30^:

(Equation 3) $$E_{pi}=E_{p1}\times \eta^{i-1}$$

(Equation 4) $$E_{p}=\sum_{i=1}^{25}E_{Pi}$$

where η = 0.97, *E*_*pi*_ is PV output (kWh) in *i* year, and *E*_*p*_ is total PV output during the life cycle (kWh). The revenue (*R*_*evenue*_) during the PV life cycle under the electricity selling strategies of “feed-into-grid surplus electricity” is calculated as follows:

(Equation 5) $$R_{evenue}=\sum_{i=1}^{25}[E_{pi}\times \left(P_{BP}+0.1\right)\times \varepsilon +E_{pi}\times \left(P_{CP}+0.1\right)\times \left(1-\varepsilon \right)]$$

where ε is the resident electricity ratio, *P*_*BP*_ is the local residential price of electricity (USD/kWh), and *P*_*CP*_ is the local commercial price of electricity (USD/kWh). The life cycle cost of the proposed PVs (*LCC*) can be found by:

(Equation 6) $$LCC=\sum_{i=1}^{25}\frac{I_{i}}{1.05^{i}}$$

where *I*_*i*_ is the investment expenditure (USD) in *i* year. Therefore, the total profit (*P*_*rofit*_), profit per capacity (*P*_*rofit*_*∗*), return on investment (*ROI*), and levelized cost of energy (*LCOE*) of the proposed solar PVs over the life cycle can be respectively calculated by:

(Equation 7) $$P_{rofit}=R_{evenue}-LCC$$

(Equation 8) $${P_{rofit}}^{*}=\frac{P_{rofit}}{C}$$

(Equation 9) $$ROI=\frac{P_{rofit}}{LCC}$$

(Equation 10) $$LCOE=\frac{\sum_{i=1}^{25}\frac{I_{i}+M_{i}+F_{i}}{{\left(1+r\right)}^{i}}}{\sum_{i=1}^{25}\frac{E_{pi}}{{\left(1+r\right)}^{i}}}$$

where *M*_*i*_ is the operations and maintenance expenditures (USD) in year *i*, *F*_*i*_ is the fuel expenditures (USD) in year *i*, *E*_*pi*_ is the electricity generation (USD) in year *i*, and *r* is the discount rate (=0.05). The calculation model is show as Table S1.

## Data Availability

• The attached Supplemental Information file includes all dataset generated or analyzed during this study. • This paper does not report original code. • Any additional information is available from the lead contact upon request.
