# Supplementary material for: Techno-economic assessment of implementing photovoltaic water villas in Maldives
Source: iScience. 2023 Apr 11;26(5):106658. doi: 10.1016/j.isci.2023.106658 (PMC10165027; doi:10.1016/j.isci.2023.106658)
Supplement: Document S1. Tables S1–S6 [file mmc1.pdf]

iScience, Volume *26*

## Supplemental information

# Techno-economic assessment of implementing photovoltaic water villas in Maldives

**Lingfei Qi, Yuan Wang, Juhuang Song, Cunhong Yin, Jinyue Yan, and Zutao Zhang**

**Table S1**. Computational model for economics of PV water villas, Related to STAR Methods.

| **Capacity** | **86.27364 (MW)** | | | |
|---|---|---|---|---|
| | **Component** | **Price** | **Unit** | **Total(USD)** |
| **FRONT FEE** | PV modules, Standard 380W module Monocrystalline Silicon price (s): Mean | 2.4998 | USD/w | 215666845 |
| | Supports/integration | 0.3335 | USD/w | 28772258.94 |
| | Inverter | 0.0435 | USD/w | 3752903.34 |
| | Wirings | 0.029 | USD/w | 2501935.56 |
| | Engineering(design, transport and assembly, install) | 0.087 | USD/w | 7505806.68 |
| | Grid connection box | 0 | USD/w | 0.00 |
| | Insurance cost | 0.005075 | USD/w | 437838.72 |
| | Grid connection fee | 0 | USD/w | 0.00 |
| | **GROSS FRONT FEE INVESTMENT** | \ | **USD** | **258637588** |
| **CHANGE INVERTER** | Inverter replacement | 10 years out of 20 years | \ | 3752903.34 |
| **YEARLY FEE** | O&M cost | 0.00435 | USD/w·year | 375290.33 |
| | Rooftop rent | \ | USD/m2/year, 10000m2 in total | \ |
| | Loan duration | 5 years | \ | \ |
| | Interest rate | 8% | \ | \ |
| | **ANNUITY** | \ | **USD/year** | **64777453.4** |
| | **YEARLY COST for first 5 years** | \ | **USD/year** | **65152743.7** |
| | **YEARLY COST for following 6-25 years** | \ | \ | **375290.33** |
| **AVERAGE PRICE PER WATT** | | | **USD/w** | **3.9717** |

**Table S2**. Techno-economic results of photovoltaic water villas on three selected sub-islands under two deployment modes, Related to STAR Methods.

| | **Ayada Maldives (USD)** | | **Angaga Island Resort (USD)** | | **JA Manafaru (USD)** | |
|---|---|---|---|---|---|---|
| | **P-demand** | **P-max** | **P-demand** | **P-max** | **P-demand** | **P-max** |
| **Annual power consumption (kWh)** | 267891 | 267891 | 84439 | 84439 | 140992 | 140992 |
| **PV capacity (kW)** | 306.18 | 1409.7 | 98.47 | 444.6 | 162.06 | 742.3 |
| **Annual solar radiation (kWh/m^2·day)** | 1852 | 1852 | 1851 | 1851 | 1936 | 1936 |
| **Annual generation (kWh)** | 442295 | 2036396 | 142169 | 641904 | 244723 | 1120932 |
| **Annual consumption/generation** | 0.605685 | 0.131551 | 0.593934 | 0.131544 | 0.576128 | 0.125781 |
| **Annual surplus/generation** | 0.394314 | 0.868448 | 0.406065 | 0.868455 | 0.423871 | 0.874218 |
| **25-year generation (kWh)** | 9951080 | 45816313 | 3198621 | 14442032 | 5505967 | 25219546 |
| **Total cost (USD)** | 1448877 | 6670855 | 465719 | 2102760 | 801668 | 3671965 |
| **Total revenue (USD)** | 210956 | 971276 | 67808 | 306161 | 116722 | 534638 |
| **Profit (USD)** | 1272612 | 4856178 | 407151 | 1529953 | 720709 | 2905399 |
| **Profit per capacity (USD)** | 4156 | 3444 | 4134 | 3441 | 4447 | 3914 |
| **ROI** | 1.2685668 | 1.0513855 | 1.2619623 | 1.0502745 | 1.3573080 | 1.1945941 |
| **Diesel electricity price (USD/kWh)** | 0.3518859 | 0.3518859 | 0.3518859 | 0.3518859 | 0.3518859 | 0.3518859 |
| **LCOE (USD/kWh)** | 0.2104758 | 0.2104758 | 0.2105895 | 0.2105895 | 0.2013435 | 0.2013435 |
| **Pay-back period** | 6 | 7 | 6 | 7 | 6 | 6 |

**Table S3**. Cash flow results of photovoltaic water villas on three selected sub-islands under two deployment modes, Related to STAR Methods.

| | **Ayada Maldives (USD)** | | **Angaga Island Resort (USD)** | | **JA Manafaru (USD)** | |
|---|---|---|---|---|---|---|
| Year | **P-demand** | **P-max** | **P-demand** | **P-max** | **P-demand** | **P-max** |
| 1 | -13918 | -152738 | -4664 | -48314 | -3202 | -14670 |
| 2 | -32921 | -322129 | -10950 | -101870 | -9413 | -43119 |
| 3 | -56328 | -505556 | -18638 | -159843 | -18248 | -83587 |
| 4 | -83524 | -700666 | -27533 | -221491 | -29360 | -171027 |
| 5 | -113956 | -905349 | -37457.9 | -286149 | -42435 | -264672 |
| 6 | 20401 | -346394 | 5625 | -109959 | 31482 | 42711 |
| 7 | 144494 | 169845 | 45418 | 52767 | 99752 | 326607 |
| 8 | 259107 | 646630 | 82170 | 203057 | 162807 | 588809 |
| 9 | 364961 | 1086973 | 116114.3 | 341859 | 221045 | 830972 |
| 10 | 454742 | 1456893 | 144896 | 458456 | 270606 | 1035269 |
| 11 | 545036 | 1832485 | 173850 | 576848 | 320284 | 1241829 |
| 12 | 628429 | 2179361 | 200591 | 686188 | 366165 | 1432598 |
| 13 | 705447 | 2499713 | 225288 | 787168 | 408539 | 1608781 |
| 14 | 776578 | 2795567 | 248097 | 880425 | 447675 | 1771494 |
| 15 | 842270 | 3068793 | 269162 | 966549 | 483819 | 1921764 |
| 16 | 902939 | 3321119 | 288616 | 1046087 | 517199 | 2060541 |
| 17 | 958969 | 3554143 | 306583 | 1119539 | 548028 | 2188704 |
| 18 | 1010714 | 3769337 | 323175 | 1187371 | 576498 | 2307063 |
| 19 | 1058501 | 3968064 | 338499 | 1250012 | 602792 | 2416366 |
| 20 | 1097730 | 4129013 | 351073 | 1300742 | 624480 | 2505421 |
| 21 | 1138485 | 4298485 | 364142 | 1354161 | 646904 | 2598636 |
| 22 | 1176121 | 4454983 | 376210 | 1403492 | 667614 | 2684717 |
| 23 | 1210877 | 4599499 | 387355 | 1449045 | 686738 | 2764209 |
| 24 | 1242973 | 4732949 | 397647 | 1491110 | 704400 | 2837614 |
| 25 | 1272612 | 4856178 | 407151 | 1529954 | 720709 | 2905399 |

**Table S4**. Calculation results of electricity cost per kilowatt hour of all PV water villas in Maldives, Related to STAR Methods.

| **Year** | **Annual power generation (APG) (kWh)** | **Discount-APG (D-APG) (kWh)** | **Yearly cost (USD)** | **Discount-yearly Cost (D-yearly cost) (USD)** |
|---|---|---|---|---|
| 1 | 1.25E+08 | 118692809 | 65152743.77 | 62050232.16 |
| 2 | 120888625.9 | 109649547.3 | 65152743.77 | 59095459.2 |
| 3 | 117261967.1 | 101295296.1 | 65152743.77 | 56281389.72 |
| 4 | 113744108.1 | 93577559.25 | 65152743.77 | 53601323.54 |
| 5 | 110331784.9 | 86447840.45 | 65152743.77 | 51048879.56 |
| 6 | 107021831.3 | 79861338.32 | 375290.33 | 280047.4254 |
| 7 | 103811176.4 | 73776664.92 | 375290.33 | 266711.8338 |
| 8 | 100696841.1 | 68155585.69 | 375290.33 | 254011.2702 |
| 9 | 97675935.87 | 62962779.16 | 375290.33 | 241915.4955 |
| 10 | 94745657.79 | 58165615.03 | 4128193.674 | 2534352.81 |
| 11 | 91903288.06 | 53733949.13 | 375290.33 | 219424.4857 |
| 12 | 89146189.42 | 49639933.96 | 375290.33 | 208975.7007 |
| 13 | 86471803.74 | 45857843.75 | 375290.33 | 199024.4768 |
| 14 | 83877649.62 | 42363912.8 | 375290.33 | 189547.1208 |
| 15 | 81361320.13 | 39136186.11 | 375290.33 | 180521.0674 |
| 16 | 78920480.53 | 36154381.45 | 375290.33 | 171924.8261 |
| 17 | 76552866.11 | 33399761.91 | 375290.33 | 163737.9296 |
| 18 | 74256280.13 | 30855018.15 | 375290.33 | 155940.8853 |
| 19 | 72028591.73 | 28504159.62 | 375290.33 | 148515.1289 |
| 20 | 69867733.98 | 26332414.13 | 4128193.674 | 1555872.779 |
| 21 | 67771701.96 | 24326134.96 | 375290.33 | 134707.5999 |
| 22 | 65738550.9 | 22472715.15 | 375290.33 | 128292.9523 |
| 23 | 63766394.37 | 20760508.28 | 375290.33 | 122183.7641 |
| 24 | 61853402.54 | 19178755.27 | 375290.33 | 116365.4896 |
| 25 | 59997800.46 | 17717516.77 | 375290.33 | 110824.2758 |
| | | 1343018227 | | 289460181.5 |
| **LCOE (USD/kWh)** | | | | |
| **0.2155296** | | | | |

**Table S5**. Calculation results of total cost and total revenue of all PV water villas in Maldives under P-max deployment mode, Related to Figure 7 and Figure 8.

| Year | Annual power generation (kWh) | D-APG (kWh) | Yearly cost (USD) | D-Yearly Cost (USD) | Accumulative D-Yearly Cost (USD) | Revenue (USD) | D-revenue (USD) | Accumulative D-Revenue (USD) | Net Cash Flow (USD) | Accumulative Net Cash Flow (USD) |
|---|---|---|---|---|---|---|---|---|---|---|
| 1 | 1.25 E+08 | 1.19E+08 | 4.49E+08 | 4.28E+08 | 4.28E+08 | 3.80E+08 | 3.62E+08 | 3.62E+08 | -6.60E+07 | -6.60E+07 |
| 2 | 1.20 E+08 | 1.10E+08 | 4.49E+08 | 4.08E+08 | 8.35E+08 | 3.69E+08 | 3.34E+08 | 6.96E+08 | -7.32E+07 | -1.40E+08 |
| 3 | 1.17 E+08 | 1.01E+08 | 4.49E+08 | 3.88E+08 | 1.22E+09 | 3.58E+08 | 3.09E+08 | 1.01E+09 | -7.93E+07 | -2.20E+08 |
| 4 | 1.14 E+08 | 9.36E+07 | 4.49E+08 | 3.70E+08 | 1.59E+09 | 3.47E+08 | 2.85E+08 | 1.29E+09 | -8.43E+07 | -3.00E+08 |
| 5 | 1.10 E+08 | 8.64E+07 | 4.49E+08 | 3.52E+08 | 1.95E+09 | 3.36E+08 | 2.64E+08 | 1.55E+09 | -8.85E+07 | -3.90E+08 |
| 6 | 1.07 E+08 | 7.99E+07 | 2.59E+06 | 1.93E+06 | 1.95E+09 | 3.26E+08 | 2.44E+08 | 1.80E+09 | 2.42E+08 | -1.50E+08 |
| 7 | 1.04 E+08 | 7.38E+07 | 2.59E+06 | 1.84E+06 | 1.95E+09 | 3.17E+08 | 2.25E+08 | 2.02E+09 | 2.23E+08 | 7.34E+07 |
| 8 | 1.01 E+08 | 6.82E+07 | 2.59E+06 | 1.75E+06 | 1.95E+09 | 3.07E+08 | 2.08E+08 | 2.23E+09 | 2.06E+08 | 2.79E+08 |
| 9 | 9.77 E+07 | 6.30E+07 | 2.59E+06 | 1.67E+06 | 1.95E+09 | 2.98E+08 | 1.92E+08 | 2.42E+09 | 1.90E+08 | 4.70E+08 |
| 10 | 9.47 E+07 | 5.82E+07 | 2.85E+07 | 1.75E+07 | 1.97E+09 | 2.89E+08 | 1.77E+08 | 2.60E+09 | 1.60E+08 | 6.30E+08 |
| 11 | 9.19 E+07 | 5.37E+07 | 2.59E+06 | 1.51E+06 | 1.97E+09 | 2.80E+08 | 1.64E+08 | 2.76E+09 | 1.62E+08 | 7.92E+08 |
| 12 | 8.91 E+07 | 4.96E+07 | 2.59E+06 | 1.44E+06 | 1.97E+09 | 2.72E+08 | 1.51E+08 | 2.91E+09 | 1.50E+08 | 9.42E+08 |
| 13 | 8.65 E+07 | 4.59E+07 | 2.59E+06 | 1.37E+06 | 1.97E+09 | 2.64E+08 | 1.40E+08 | 3.05E+09 | 1.38E+08 | 1.08E+09 |
| 14 | 8.39 E+07 | 4.24E+07 | 2.59E+06 | 1.31E+06 | 1.98E+09 | 2.56E+08 | 1.29E+08 | 3.18E+09 | 1.28E+08 | 1.21E+09 |
| 15 | 8.14 E+07 | 3.91E+07 | 2.59E+06 | 1.24E+06 | 1.98E+09 | 2.48E+08 | 1.19E+08 | 3.30E+09 | 1.18E+08 | 1.33E+09 |
| 16 | 7.89 E+07 | 3.62E+07 | 2.59E+06 | 1.19E+06 | 1.98E+09 | 2.41E+08 | 1.10E+08 | 3.41E+09 | 1.09E+08 | 1.44E+09 |
| 17 | 7.66 E+07 | 3.34E+07 | 2.59E+06 | 1.13E+06 | 1.98E+09 | 2.33E+08 | 1.02E+08 | 3.52E+09 | 1.01E+08 | 1.54E+09 |
| 18 | 7.43 E+07 | 3.09E+07 | 2.59E+06 | 1.08E+06 | 1.98E+09 | 2.26E+08 | 9.41E+07 | 3.61E+09 | 9.30E+07 | 1.63E+09 |
| 19 | 7.21 E+07 | 2.85E+07 | 2.59E+06 | 1.02E+06 | 1.98E+09 | 2.20E+08 | 8.69E+07 | 3.70E+09 | 8.59E+07 | 1.72E+09 |
| 20 | 6.99 E+07 | 2.63E+07 | 2.85E+07 | 1.07E+07 | 1.99E+09 | 2.13E+08 | 8.03E+07 | 3.78E+09 | 6.96E+07 | 1.78E+09 |
| 21 | 6.78 E+07 | 2.43E+07 | 2.59E+06 | 9.29E+05 | 1.99E+09 | 2.07E+08 | 7.42E+07 | 3.85E+09 | 7.32E+07 | 1.86E+09 |
| 22 | 6.57 E+07 | 2.25E+07 | 2.59E+06 | 8.85E+05 | 1.99E+09 | 2.00E+08 | 6.85E+07 | 3.92E+09 | 6.76E+07 | 1.93E+09 |
| 23 | 6.38 E+07 | 2.08E+07 | 2.59E+06 | 8.43E+05 | 1.99E+09 | 1.94E+08 | 6.33E+07 | 3.98E+09 | 6.25E+07 | 1.99E+09 |
| 24 | 6.19 E+07 | 1.92E+07 | 2.59E+06 | 8.03E+05 | 2.00E+09 | 1.89E+08 | 5.85E+07 | 4.04E+09 | 5.77E+07 | 2.05E+09 |
| 25 | 6.00 E+07 | 1.77E+07 | 2.59E+06 | 7.64E+05 | 2.00E+09 | 1.83E+08 | 5.40E+07 | 4.10E+09 | 5.33E+07 | 2.10E+09 |
| | | | **Total cost** | **1996277114** | | **Total revenue** | **4.095E+09** | | | |

**Table S6**. Overall techno-economic benefits of all photovoltaic water villas in Maldives under P-max deployment mode, Related to STAR Methods.

| **Deployment mode** | P-max |
|---|---|
| **Annual power consumption (kWh)** | 1.6E+07 |
| **PV capacity (kW)** | 86273.6 |
| **Annual solar radiation (kWh/m^2·day)** | 1852 |
| **Annual generation (kWh)** | 1.25E+08 |
| **Annual consumption/generation** | 0.131552 |
| **Annual surplus/generation** | 0.868448 |
| **25-year generation (kWh)** | 2.8E+09 |
| **Total cost (USD)** | 4.08E+08 |
| **Total revenue (USD)** | 59442123 |
| **Profit (USD)** | 3E+08 |
| **Profit per capacity (USD)** | 3444.83 |
| **ROI (return on investment)** | 1.05139 |
| **Diesel electricity price (USD/kWh)** | 0.35189 |
| **LCOE (USD/kWh)** | 0.21048 |
| **Pay-back period** | 7 |
